# The Role of Female Physicians in Psychosomatic Medicine: Opportunities and Challenges

**DOI:** 10.1089/whr.2023.0070

**Published:** 2024-01-11

**Authors:** Caroline Rometsch

**Affiliations:** Department of Experimental and Clinical Medicine, University of Florence, Firenze, Italy.

**Keywords:** epidemiology, female physicians, mental health, occupational health, psychosomatic medicine

## Abstract

**Background:**

Female physicians are in some cases preferred by patients due to their sex-related characteristics such as softness and empathy. Psychosomatic medicine presents a compelling working environment due to its holistic approach.

**Methods:**

This brief review synthesizes the challenges encountered by female physicians in psychosomatic medicine and outlines potential strategies for overcoming these barriers.

**Results:**

The presence of female role models may constitute a crucial advancement in this process. There exists a pressing demand for specialized clinical and scientific programs in psychosomatic medicine at both national and international levels. Such programs, offered by universities and ministries, as well as comprehensive training initiatives, are indispensable in fostering the next generation of females in psychosomatics. Leading journals can lend their support by publishing special issues dedicated to female physicians.

**Conclusion:**

Strengthening female physicians throughout all positions in psychosomatic medicine can contribute ultimately to the improvement of patient care.

## The Role of Female Physicians in Medicine

While it was debated half a century ago whether female physicians were preferred by female patients, it is now well-established that the sex of both the physician and patient significantly influences their interaction.^1^ Some female patients prefer to communicate with female physicians instead of male physicians due to cultural reasons,^2^ hereby speaking more about biomedical and psychosocial difficulties, making more positive statements, and focusing more often on partnership problems.^3^ However, medicine was dominated by male physicians for centuries.^4^ Investigations into clinical practice have unveiled noteworthy distinctions concerning gender interactions up to now,^5^ wherein female physicians tend to offer a higher frequency of preventive health care services and psychosocial counseling. In contrast, their male counterparts exhibit a greater emphasis on medical history assessment and physical examinations.^1^ Furthermore, with regard to medical specialization, gender disparities manifest as fewer females opt for surgical and internal medicine specialties. This phenomenon can be attributed to a multifaceted construct encompassing factors such as workload and childcare responsibilities.^6^ Gender disparities in the medical field are, in part, perpetuated by the presence of gender blindness and stereotyped preconceptions.^7^ Historically, and due to specific cultural reasons (*e.g.*, negative stereotypes of female scientists,^8^ family interferences with work,^9^ educational unequal opportunities^10^), females in all scientific disciplines, not just in medicine, have had to surmount considerable obstacles and engage in persistent struggles to assert themselves in academic fields. These women, including notable figures as Marie Skłodowska Curie, Rosalind Franklin, and Ada Hopper, achieved global recognition. Only 120 years ago, Dr. Hermine Heusler-Edenhuizen became the first woman in Germany to pass her medical exams and earn the right to practice medicine.^11^ Female physicians, often associated with empathy and nurturing qualities,^12^ are of principal importance in all disciplines. These works aim to synthesize obstacles for female physicians nowadays and demonstrate opportunities for female physicians in psychosomatics.

## Psychosomatic Medicine

Psychosomatic medicine is an integral aspect in medicine,^13^ adhering to a biopsychosocial model of care.^13^ It is recognized as an all-encompassing discipline that provides integrated care, thereby transcending the dichotomy of body and mind to treat individuals holistically.^14^ Not to be conflated with psychiatry or liaison-psychiatry,^15^ psychosomatic medicine has a long-standing tradition with roots dating back to ancient Greece, and further enriched by influential figures such as Heinroth,^16^ Alexander,^17^ and Engel.^18^ Globally, psychosomatic medicine is not firmly established as an independent institution. Physicians with a specific interest in psychosomatic medicine can be found within somatic-oriented disciplines or in psychiatry. One major challenge for the field of psychosomatic medicine is the ongoing endeavor to define its position and gain acceptance among various disciplines. Despite the fast rise of psychosomatics in Western countries and Asia^19^ based on the activities of the Japanese Professor Yujiro Ikemi in Fukuoka,^20^ this process was not followed by other countries in the same manner. In Africa and Egypt, there exists only little knowledge on psychosomatic medicine.^19^ In a global context, owing to significant historical milestones,^21^ psychosomatic medicine achieved its independent status as a specialty and institution primarily in Germany.^22^ Patients with functional disorders (*e.g.*, irritable bowel syndrome, chronic pain, chronic fatigue syndrome, or fibromyalgia), eating disorders, the so-called “somatopsychic disorders” (*e.g.*, psychocardiology, psychooncology), affective disorders, personality disorders, and trauma disorders are treated after a consultation with a physician of psychosomatic medicine in one of around 223 hospitals for psychosomatic medicine, departments of psychosomatic medicine at general or psychiatric hospitals, or academic institutions in Germany.^21,22^

Since statistical analysis on physicians in psychosomatic medicine is primarily available for Germany, this article will provide an overview focusing on this European nation. Current statistics for 2021 indicate a total of 416,120 physicians, a steady increase compared to data from 1990.^23^ Nearly half of these (201,951) are females. The majority of physicians specialize in internal medicine, which, in some cases, incorporates psychosomatics (*e.g.*, Tübingen and Heidelberg).^22^ Physicians who opt for specialization in psychosomatic medicine and psychotherapy constitute a minority with ∼5,000 individuals.^23^ According to the statistics, half of all physicians in psychosomatic medicine are females, with a count of 2,322.^23^ Historically, the progress toward gender equality in the psychosomatic field has followed a trajectory similar to that observed in other specialties, where the proportion of female representation has significantly increased over the past few decades.^21,24^

## Challenges for Female Physicians in Psychosomatic Medicine

Despite the sex balance among practicing physicians in psychosomatic medicine nowadays, career advancement statistics in Germany reveal a striking disparity. Although females constitute half of the physicians in this field, they hold only a third of all professorships in medicine.^25^ In line, according to United Nations Educational, Scientific and Cultural Organization (UNESCO), females constitute a minority in research globally, with the highest proportions found in South-Eastern Europe (49%), followed by the Caribbean, Central Asia, and Latin America (44%).^26^ Recent studies indicate that despite the implementation of programs aimed at enhancing female's participation in science *via* diversity and equity initiatives, sex bias remains in medicine, affecting female's grant applications, remuneration, and overall success.^27^

Several variables may explain this complex phenomenon. Females are generally underrepresented in science, which may be attributed to the significant yet inadequate presence of female career role models. Early adolescent females demonstrated improved attitudes toward science when exposed to females in scientific roles.^28^ Unfortunately, such role models are still scarce in psychosomatic medicine. One prominent barrier to career progression are domestic and family responsibilities of female physicians^29^ and less work control compared to male physicians.^30^ Male physicians work more hours; besides, they are more willing to marry and have children, which spur their career options, compared to female physicians.^31^ Despite increased role complexity was related to stress for both male and female physicians^32^; nowadays, female physicians are more often responsible for family duties than male physicians.^33^ However, a change in work–life balance with more willingness to parent in males can be observed.^34^ Additionally, earning gaps between female and male physicians are found, even in the last 20 years.^35^ Female physicians show higher rates of mental illnesses^36^ (*e.g.*, depression^37^) and are at higher risk of suicide.^38^ Regrettably, instances of sexual harassment persist in medicine and in science in general.^27^ Further investigations show that female physicians face inequalities which do not disappear with age or seniority.^39^ These findings apply to medicine in general but are also valid for psychosomatic medicine.

## Opportunities for Young Female Physicians in Psychosomatic Medicine

In alignment with strategies for enhancing women's participation in science, various resources for young female physicians and scientists are proposed at international and national levels by leading organizations such as the World Health Organization,^40,41^ European Commission,^42^ and UNESCO.^26^ The promotion of females in science must commence during early education, as asserted by the European Council in 2018.^43^ Some progress has been observed though it remains insufficient.^44^ However, data specific to psychosomatic medicine is lacking.

Universities can actively support young female students interested in pursuing a career in medicine in general, and in particular, in psychosomatic medicine. The Research Partnership on Women in Science Careers offers advice on overcoming barriers in six thematic areas: career progression, mentorship, work–life balance, pathways to leadership, pay equity, and advocacy for change.^45^ These recommendations might support overcoming some challenges. Addressing family responsibilities, part-time models in medicine are urgently required which meanwhile become more and more prominent in psychosomatic medicine, leading psychosomatics to be an attractive discipline for female physicians. While some support exists, increased efforts are needed to foster the combination of research activities with clinical work.

Networks in psychosomatics contribute to a higher engagement of female physicians in psychosomatic (*e.g.*, International College of Psychosomatic Medicine [ICPM], or European Association of Psychosomatic Medicine [EAPM]), hereby combining clinical and scientific work. In the European psychosomatic field, special interest groups, particularly for early career researchers (ECRs), support collaboration and the exchange of ideas. National initiatives such as the German *Perspective Psychosomatic*, the Greek *International Society for Research of Interplay between Mental and Somatic Disorders*, and the Spanish *Ibero-American Society of Psychosomatic Studies*, unite the ECR psychosomatic community. Annual conferences, such as those held by the EAPM, ICPM, and the German Psychosomatic Conference, can provide inspiration and a sense of community. Lastly, scientific journals can aid the advancement of female physicians in psychosomatic medicine. All those opportunities seem to be best achieved through a clear role of policy changes and institutional support in facilitating improvements for female physicians.

## Clinical-Scientific Programs

In Germany, universities offer support programs for women in medicine,^46^ setting a good example for other European countries. “Clinician scientist” programs, which provide personalized education plans combining clinical work and research,^47,48^ can be a first step toward competitiveness of female physicians in this field. Though these programs are seldom found in psychosomatic medicine, they could serve as a template for all female physicians in this discipline, potentially offering regular work, mentorship, and ideally, female role models as supervisors.^49^ Grants that include comprehensive training and structured research programs for ECRs are, without question, one of the most significant opportunities a female physician can take in overcoming the gender gap. The Encompassing Training in fUnctional Disorders across Europe (ETUDE) program (https://etude-itn.eu/, No. 956673), where some ECR positions are held by female physicians, is a prime example. It aims to train ECRs in functional disorders within a 3-year program. As a training network, ETUDE uniquely integrates clinical aspects of diagnostic procedure, stigmatization, and patientcare into its framework, allowing female physicians to acquire essential skills for clinical praxis. Functional disorders as an umbrella term refers to persistent somatic symptoms without reproducibly pathophysiological mechanisms.^50^ This Marie Skłodowska-Curie Innovation Training Network serves as an essential platform for facilitating interconnections among female scientists and for disseminating knowledge across successive generations, thereby acting as a paradigmatic example to overcome the existing gender-gap. During their participation in the ETUDE program, female physicians can engage in clinical observations, accompany patient consultations, and collaborate with experienced clinicians. These experiences enable them to develop a deeper understanding of functional disorders and their clinical manifestations. Furthermore, this program significantly enhances the understanding of functional disorders within the medical community and will contribute to provide evidence-based guidelines for patient management, which will be assessable globally.

The ETUDE program holds particular relevance for female physicians in Germany as it provides a unique opportunity to bridge the gender gap in the field of psychosomatic medicine. By offering comprehensive training, research experience, and clinical insights, ETUDE equips female physicians with the skills and knowledge needed to expand their roles and responsibilities. With this innovative training, female physicians can enhance their competence, meeting German governmental requirements for university positions and expanding their career opportunities. Furthermore, it serves as a model for integrating clinical practice and research, thus empowering female physicians to take on more significant roles in addressing complex health care challenges.

## Conclusion

Strengthening female physicians in psychosomatic medicine leads to the improvement of patient care and research advances, given the unique perspectives and approaches that female physicians can bring to the field. To address existing disparities, there is need for the implementation of supportive policies and programs within the field of psychosomatic medicine at both national and international levels. The ETUDE initiative, a part of the Marie Skłodowska-Curie Innovation Training Network, exemplifies an optimal model for internationally advancing the careers of female physicians and scientists within the domain of psychosomatic medicine. Additionally, implementing strategies such as providing increased opportunities for part-time work or flexible schedules, promoting mentorship programs with female role models, and fostering networking and community support are essential in overcoming barriers.

## Abbreviations Used

EAPM: European Association of Psychosomatic Medicine
ECRs: early career researchers
ICPM: International College of Psychosomatic Medicine
